# Life Stress and Dementia Risk in Adults with Down Syndrome

**DOI:** 10.1093/geroni/igaf122.3619

**Published:** 2025-12-31

**Authors:** Alexandra Linker, Sigan Hartley, Shahid Zaman, Luciana Fonseca

**Affiliations:** Washington State University, Spokane, Washington, United States; University of Wisconsin - Madison, Madison, Wisconsin, United States; University of Cambridge, Cambridge, England, United Kingdom; Krieger Klein Alzheimer’s Research Center and the Rutgers Brain Health Institute, Piscataway, New Jersey, United States

## Abstract

Down syndrome (DS) is the most common genetic cause of intellectual disability, resulting from a triplication of chromosome 21. Individuals with DS are at a higher risk of developing Alzheimer’s Disease (AD) compared to the general population. Previously, exposure to stress has been suggested as a risk factor for dementia and AD in the general population. However, the role of life stress in the timing of AD in DS is underinvestigated. Using the Alzheimer Biomarkers Consortium - Down Syndrome (ABC-DS) dataset, we analyzed the association between baseline life stressors, and later cognitive decline and dementia at 16 or 32-month follow-up. We found that a higher baseline sum of stress events was associated with greater risk of conversion from baseline stable cognition to mild cognitive impairment (MCI) or dementia at follow up, even when controlling for age, sex, premorbid level of intellectual disability, site, clinical latency between assessments, and the presence of anxiety or depression (odds ratio = 1.53, p = 0.021). Additionally, the death of a loved one at baseline, when rated as having a negative or complex impact, was associated with a decline of more than 6 points on the Block Design test (p = 0.009), compared to those without this experience. These results suggest that life stressors may predict cognitive decline and dementia in adults with DS, highlighting a potential target for treatment or intervention. Results also suggest that there may be similar risk factors for AD in adults with DS as those found in the general population.

